# High vs. Low Radiation Dose of Concurrent Chemoradiotherapy for Esophageal Carcinoma With Modern Radiotherapy Techniques: A Meta-Analysis

**DOI:** 10.3389/fonc.2020.01222

**Published:** 2020-08-04

**Authors:** Xin Sun, Lei Wang, Yang Wang, Jingjing Kang, Wei Jiang, Yu Men, Zhouguang Hui

**Affiliations:** ^1^Department of Radiation Oncology, National Cancer Center/National Clinical Research Center for Cancer/Cancer Hospital, Chinese Academy of Medical Sciences and Peking Union Medical College, Beijing, China; ^2^State Key Laboratory of Oncology in South China, Department of VIP Region, Sun Yat-sen University Cancer Center, Collaborative Innovation Center for Cancer Medicine, Guangzhou, China; ^3^Department of Radiation Oncology, National Cancer Center/National Clinical Research Center for Cancer/Cancer Hospital and Shenzhen Hospital, Chinese Academy of Medical Sciences and Peking Union Medical College, Shenzhen, China; ^4^Department of VIP Medical Services, National Cancer Center/National Clinical Research Center for Cancer/Cancer Hospital, Chinese Academy of Medical Sciences and Peking Union Medical College, Beijing, China

**Keywords:** esophageal carcinoma, chemoradiotherapy, radiation dose, high dose, low dose, meta-analysis

## Abstract

**Purpose:** The aim of this study was to evaluate the clinical benefit of different radiation doses in concurrent chemoradiotherapy (CCRT) for esophageal carcinoma using modern radiotherapy techniques.

**Methods:** A systematic review was conducted by screening PubMed, EMBASE, Cochrane Central Register of Controlled Trials, SCOPUS, Wanfang, and Chinese National Knowledge Infrastructure (CNKI) databases with prespecified searching strategy. Studies which compared high radiation dose group with low-dose radiation group using modern radiotherapy techniques for esophageal cancer patients in CCRT were identified. The hazard ratios (HR) for overall survival (OS) and the odds ratios (OR) for local–regional failure (LRF), distant metastasis (DM), and toxicities were considered as the outcomes of interest. R 3.6.2 software was used for statistical analysis.

**Results:** Twelve studies involving 10,896 patients were included for analyses. The results showed that the high-dose group had better OS (HR = 0.79, 95% CI = 0.70–0.90, *P* = 0.0004) and the local–regional control (OR = 0.59, 95% CI = 0.46–0.76, *P* < 0.0001), especially for patients who were diagnosed with squamous cell carcinoma (SCC). The subgroup analyses further indicated that ≥ circa 60 Gy can significantly improve the OS (HR = 0.73, 95% CI = 0.68–0.80, *P* < 0.0001) as well as the local–regional control (OR = 0.54, 95% CI = 0.40–0.74, *P* < 0.0001) as compared with < circa 60 Gy. Another subgroup analysis comparing ≤ 50.4 Gy with > 50.4 Gy showed no substantial difference in OS (HR = 0.98, 95% CI = 0.93–1.03, *P* = 0.43). In addition, there are no significant differences between the two groups in grade 3–5 radiation pneumonitis (OR = 1.05, 95% CI = 0.54–2.05, *P* = 0.89), grade 3–5 radiation esophagitis (OR = 1.40, 95% CI = 0.93–2.11, *P* = 0.11), treatment-related death (OR = 1.60, 95% CI = 0.70–3.66, *P* = 0.27), and DM (OR = 1.21, 95% CI = 0.92–1.59, *P* = 0.17).

**Conclusions:** For esophageal carcinoma receiving CCRT with modern radiation techniques, evidence suggested that high-dose radiotherapy, especially ≥circa 60 Gy, had potentials to improve the OS and local–regional control without increase in severe toxicities when compared with low-dose radiotherapy. The result needs to be confirmed by randomized clinical trials.

## Introduction

Definitive concurrent chemoradiotherapy (CCRT) is the standard treatment modality for inoperable locally advanced esophageal carcinoma, and patients refused surgery (1–4). Local recurrence within the gross tumor volume (GTV) is the most common treatment failure modality (5–7). Escalation of the radiation dose can reduce the local recurrence, which is very likely to associate with better overall survival (OS). However, the optimal radiotherapy dose for inoperable esophageal carcinoma patients undergoing CCRT is still controversial. INT 0123 randomized controlled trial (RCT) (8), disclosed 20 years ago using the two-dimensional conventional radiotherapy (2D-CRT) technique, showed that the higher radiation dose (64.8 Gy) produce no extra benefit on survival but rather a higher treatment-related mortality rate compared to the standard dose (50.4 Gy) for definitive CCRT. Nowadays, 3-dimensional conformal radiation therapy (3D-CRT) and intensity-modulated radiation therapy (IMRT) have become widely used and are able to deliver a higher dose to GTV without causing more toxicities (9). This study evaluates the benefit and risk of high vs. low radiation dose using modern techniques on survival, local control, distant metastasis (DM), and toxicities of patients with locally advanced esophageal carcinoma undergoing CCRT.

## Materials and Methods

### Search Strategy

A literature search was performed systematically for the following databases: PubMed, EMBASE, Cochrane Central Register of Controlled Trials, SCOPUS, Wanfang, and Chinese National Knowledge Infrastructure (CNKI). All trials published prior to December 31, 2019, were targeted. Computer retrieval was performed using the following retrieval language: [(“esophageal”[Title]) or (“oesophageal”[Title]) or (“esophagus”[Title])] and [(“tumor”[Title]) or (“cancer”[Title]) or (“carcinoma”[Title]) or (“neoplasm”[Title]) or (“neoplasms”[Title])] and [(“chemoradiation”[Title]) or (“chemoradiotherapy”[Title]) or (“radiochemotherapy”[Title]) or (“chemo-irradiation”[Title]) or (“chemo-radiotherapy”[Title])] and (“dose”[Abstract]). To ensure the integrity and comprehensiveness, manual searches of reference lists were also performed.

### Inclusion and Exclusion Criteria

**Figure 1:**
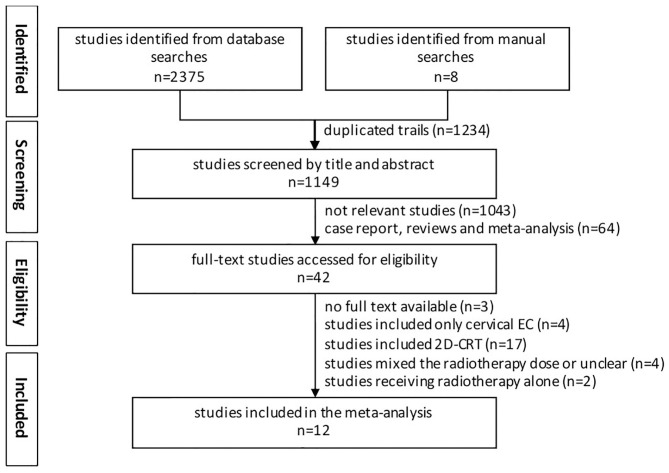
Flow chart showing inclusion and exclusion of studies.

Studies included in our analyses had to meet the following criteria: (1) Clinical trials must compare high-dose radiotherapy (HD-RT) to low-dose radiotherapy (LD-RT); (2) Studies on initially diagnosed esophageal carcinoma received external beam radiotherapy with concurrent chemotherapy; (3) Data on OS had to be reported; (4) The language of publication abstract was limited to English; (5) All the randomized controlled trials (RCTs) and nonrandomized controlled trials (NRCTs) were eligible. Studies were excluded as follows: (1) Duplicate studies; (2) Incomplete information or data; (3) Radiotherapy delivered by 2D-CRT; (4) Patients treated by brachytherapy; (5) Review, meta-analysis, case report, basic research, ongoing clinical trial; (6) Included cervical esophageal carcinoma only; and (7) Full text not available. The workflow is shown in Figure 1.

### Methodological Quality Assessment

The quality of RCT was assessed using the modified Jadad score (10). Total scores from 1 to 3 indicate poor quality while scores from 4 to 7 stand for high quality. The NRCTs were evaluated according to the 9-star Newcastle–Ottawa Scale (NOS) (available from: http://www.ohri.ca/programs/clinical_epidemiology/oxford.htm). The quality categories were defined as follows: high quality (score 7–9), medium quality (score 4–6), and low quality (score less than 4). The quality of included studies was independently assessed by two reviewers.

### Data Extraction

For each study, the following data was extracted: first author's name, year of publication, the author's country, type of study, study period, age, sample size, tumor staging and location, histology, radiation technology, radiation dose, chemotherapy regimens, and follow-up time; the outcomes including HR of OS, the observed frequencies of local–regional failure (LRF), DM, and incidence of toxicities. Two investigators (X Sun and L Wang) independently extracted the data and reached a consensus on all variables. Detailed information on all included studies is presented in Table 1.

**Table 1:** Characteristics of all clinical trials included in the meta-analysis.

**Studies**	**Country**	**Study** **Design**	**Inclusion** **Period**	**Age** **(Median, Range)**	**Sample** **Size** **(LD/HD)**	**Stage**	**Location** **(Cervical/Upper/** **Middle/Lower/** **GJ/Unknown)**	**Histology** **(SCC/AC/** **Others)**	**Radiation** **Technology**	**Radiotherapy Dose**		**Concurrent** **Chemotherapy**	**Follow-Up Time** **(Median, Range)**	**Studies'** Quality*
										**LD-RT**	**HD-RT**			
(11)	China	Prospective	2007.1–2007.12	57, 46–68	24/20	I–III^*^	44 (cervical and upper)	44/0/0	IMRT	60 Gy/2 Gy	63.9 Gy/2.27 Gy	PF	36 m, –	5
(12)	France	Retrospective	2003.1–2006.12	65, 42–81	60/83	I–IVa^*^	4/29/52/56/2	113/30/0	3D-CRT	38–50.4 Gy/ 1.8–2 Gy	50.7–72 Gy/ 1.8–2.5 Gy	Cisplatin/5- FU/taxane	20.8 m, 2.8–92.4 m	6
(13)	USA	Retrospective	1998.5–2012.5	68, 30–89	137/56	I–IV*/^**^	0/66/40/87/0/0	193/0/0	3D-CRT/IMRT	41.4–50.4 Gy/1.6–2.4 Gy	52.2–66 Gy/1.6–2.4 Gy	Platin-/taxane-based	32.4 m, 2.5–161.3 m	6
(14)	USA	Retrospective	2004–2012	69, 26–90	3821/3033	I–III*/^**^	859 (cervical and upper)/1549/ 3693/753/0	3049/ 3602/203	3D-CRT/IMRT	≤ 50.4 Gy/–	>50.4 Gy/–	–	–	6
(15)	Taiwan area	Retrospective	2008–2013	–	324/324	I–IVa	40/608 (/upper/middle/ lower/GJ/ unknown)	648/0/0	CRT/IMRT	50–50.4 Gy/–	≥60 Gy/–	–	–	5
(16)	Taiwan area	Retrospective	2006.1–2014.12	57, 29–92	1134/927	I–III	2061 (thoracic)	2061/0/0	IMRT	45–59.4 Gy/1.8 Gy	60–72 Gy/1.8 Gy	–	26.4 m, 19.4–33.2 m	7
(17)	China	Retrospective	2010–2016	68, 36–81	63/74	I–III^**^	0/29/57/51/0/0	137/0/0	3D-CRT/IMRT	50–50.4 Gy/1.8–2 Gy	≥59.4 Gy/1.8–2 Gy	5-FU based	27.5 m, 6.4–79.5 m	7
(18)	Korea	Retrospective	1994.2–2013.5	67, 30–86	120/116	II–III^**^	16/58/113/49/0	230/6/0	3D-CRT/IMRT/TOMO	<60 Gy/1.8–2 Gy	≥60 Gy/1.8–2 Gy	PF/5-FU/ Cisplatin	19.4 m, 2.2–164.7 m	6
(19)	Taiwan area	Retrospective	2007–2015	56, 40–83	42/42	I–III^**^	38/28/18 (cervical and upper/ middle/lower)	84/0/0	IMRT/VMAT	44–50.4 Gy/–	52.2–70 Gy/–	–	23.2 m, –	6
(20)	China	Retrospective	2004–2013	–	190/190	I–IVa^***^	137/243 (cervical and upper/ middle and lower)	380/0/0	3D-CRT/IMRT	50.4–54 Gy/1.8 Gy	60 Gy/2 Gy	PF/TP	87 m, –	8
(21)	China	Retrospective	2009.1–2014.3	67, 46–79	43/37	II–III^**^	0/14/31/35/0/0	80/0/0	3D-CRT/IMRT	50.4–56 Gy/1.8–2 Gy	59.4–64.8 Gy/1.8–2 Gy	PF/TP/S-1	54 m, −91 m	6
(22)	Taiwan area	Retrospective	2011–2015	59, –	18/18	I–III^**^	–	36/0/0	IMRT	50 Gy/1.8–2 Gy	60 Gy/1.8–2 Gy	–	10 m, 2–82 m	6

LD, low dose; HD, high dose; RT, radiotherapy; SCC, squamous cell cancer; AC, adenocarcinoma; 3DRT, three-dimensional conformal radiotherapy; IMRT, intensity-modulated radiotherapy; VMAT, volume-modulated arc therapy; TOMO, helical tomotherapy; 5-FU, 5-fluorouracil; PF, cisplatin + 5-fluorouracil; PF, cisplatin + 5-fluorouracil; TP, paclitaxel + cisplatin;

* staged according to the sixth edition of the American Joint Committee on Cancer (AJCC) staging manual for esophageal carcinoma;

** staged according to the seventh edition of the American Joint Committee on Cancer (AJCC) staging manual for esophageal carcinoma;

*** *staged according to the eighth edition of the American Joint Committee on Cancer (AJCC) staging manual for esophageal carcinoma; m, month*.

### Statistical Analyses

This meta-analysis was performed with the software of R Version 3.6.2. Survival rates from Kaplan–Meier curves were read using Engauge Digitizer version 4.1 (available from: http://digitizer.sourceforge.net/), and the HR were then derived using the calculation spreadsheet appended to Tierney's paper. The inversed-error-weighted meta-analyses were conducted for outcomes of interest. The statistical heterogeneity of each study was assessed by *I*^2^ (23). If *I*^2^ ≤ 50% which indicated no significant heterogeneity among studies, a fixed-effects model was used to synthesize hazard ratio (HR) and odds ratio (OR); otherwise, a random-effects model was employed. Potential publication bias was evaluated by Egger's and Begg's test (24, 25).

## Results

### Study Characteristics

The characteristics of 12 studies were presented in Table 1 (11–22), which consisted of 1 RCT (11), four population-based propensity-score matched analyses (14, 15, 20, 22), and seven retrospective control studies (12, 13, 16–19, 21). There were nine studies that came from Asian countries (including one from Korea, four from Taiwan area, and three from China) and three studies from western countries (including two from USA and one from France). One lakh eight hundred and ninety-six patients with esophageal carcinoma were included in the final meta-analysis, with a follow-up time range of 2.0–164.7 months. The median age at diagnosis ranged from 56 to 69 years. 64.7 and 33.4% of patients were diagnosed with squamous cell carcinoma (SCC) and adenocarcinoma (AC), respectively. Nine studies enrolled patients with SCC only (11, 13, 15–17, 19–22), while for the other three studies both SCC and AC were eligible. Tumor nodal metastasis (TNM) stage of the patients ranged from I to IV. All patients received modern radiation techniques, including 3D-CRT, IMRT, VMAT, and TOMO therapy. The total delivered radiation dose ranged from 38 to 72 Gy, and the radiation dose per fraction ranged from 1.6 to 2.5 Gy. Five thousand and nine hundred and seventy-six patients received a total dose of 38–60 Gy in the LD-RT group and 4,920 patients received a total dose of 50.4–72 Gy in the HD-RT group.

### Assessment of the Studies' Quality

The details of assessment regarding the RCTs and NRCTs are shown in Supplementary Tables 1, 2. The modified Jadad score of the only RCT was 5 of 7, exhibiting that the quality was high. The 9-star NOS scores of all included NRCTs ranged between 5 and 7, and the average score was 7.1. Therefore, the overall quality of the included studies was sufficient.

### Effect of Radiation Dose on Survival

**Figure 2:**
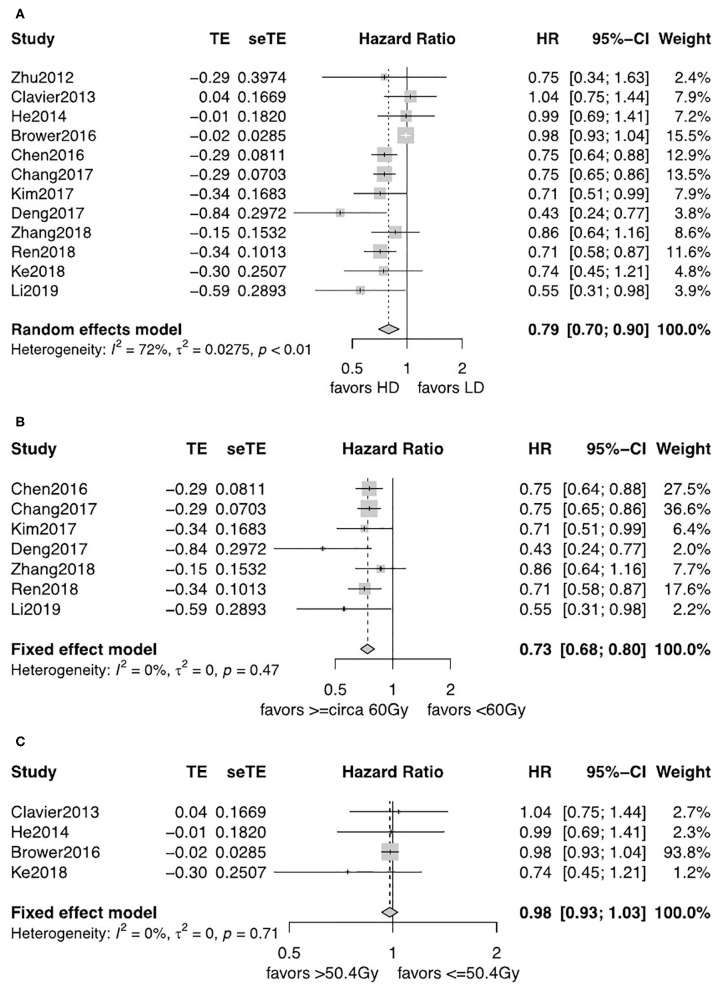
**(A)** Forest plot of hazard ratio (HR) of OS in comparison of LD-RT group to HD-RT group; Publication bias: Begg's test, *P* = 0.89. **(B)** Forest plot of hazard ratio (HR) of OS in comparison of LD-RT subgroup (<circa 60 Gy) to HD-RT subgroup (≥circa 60 Gy); Publication bias: Begg's test, *P* = 0.051; Egger's test, *P* = 0.13. **(C)** Forest plot of hazard ratio (HR) of OS in comparison of LD-RT subgroup (≤ 50.4 Gy) to HD-RT subgroup (>50.4 Gy); Publication bias: Begg's test, *P* = 0.17; Egger's test, *P* = 0.61. CI, confidence interval; HD, high dose; LD, low dose; RT, radiotherapy.

All studies reported an OS Kaplan–Meier curve stratified by the LD-RT and HD-RT groups. There was evident heterogeneity for the results among the 12 studies, and a random-effects model was used. There was statistically significant benefit on OS in the HD-RT group when compared with the LD-RT group (pooled HR = 0.79, 95% CI = 0.70–0.90, *P* = 0.0004; Figure 2A). No publication bias was detected for the pooled estimate survival, using the Begg's test. Moreover, different cutoffs were applied in defining high and low radiation dose patients. We categorized the 12 studies into subgroups based on the threshold of <circa 60 Gy vs. ≥circa 60 Gy (due to the difference in the fraction dose radiotherapy, the thresholds of 59.4 and 60 Gy were included in the circa 60-Gy subgroup) and ≤ 50.4 vs. >50.4 Gy. Seven studies were included in the subgroup analysis of <circa 60 Gy (range: 45–59.4 Gy) vs. ≥circa 60 Gy (range: 60–72 Gy) (15–18, 20–22). Four studies were included in the subgroup analysis of ≤ 50.4 Gy (range: 38–50.4 Gy) vs. > 50.4 Gy (range: 38–50.4 Gy) (12–14, 19). As there was only one study comparing 60 with 63.9 Gy, we did not perform a pooled analysis (11). The heterogeneity of subpopulations was reduced where cutoffs were a major source. The fixed-effects model was applied. As shown in Figure 2B, the patients who received ≥circa 60 Gy gain substantial survival benefits when compared with patients that received <circa 60 Gy (pooled HR = 0.73, 95% CI = 0.68–0.80, *P* < 0.0001). For subgroups comparing ≤ 50.4 with >50.4 Gy, no significant OS benefits was observed (pooled HR = 0.98, 95% CI = 0.93–1.03, *P* = 0.43; Figure 2C).

**Figure 3:**
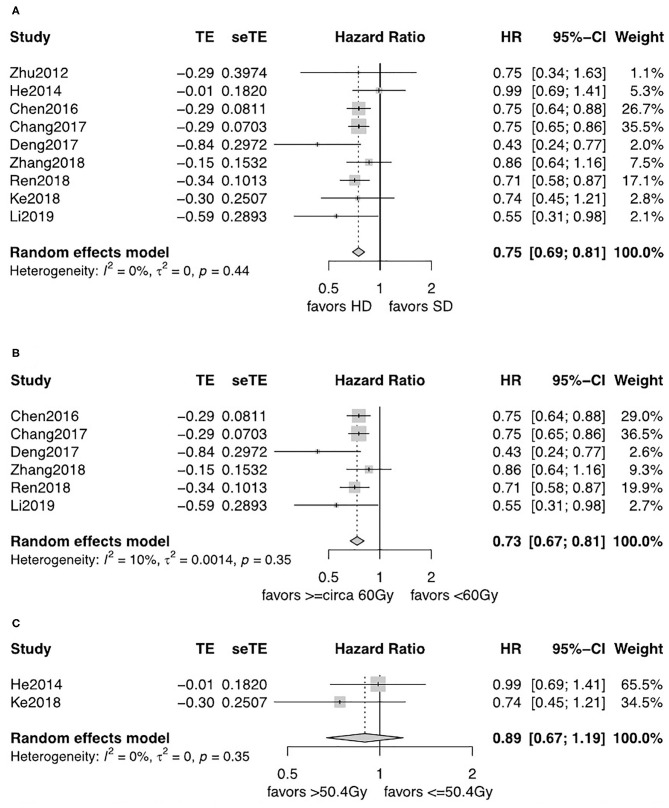
**(A)** Forest plot of hazard ratio (HR) of OS in comparison of LD-RT group to HD-RT group for SCC; Publication bias: Begg's test, *P* = 0.21; Egger's test, *P* = 0.56. **(B)** Forest plot of hazard ratio (HR) of OS in comparison of LD-RT subgroup (<circa 60 Gy) to HD-RT subgroup (≥circa 60 Gy) for SCC; Publication bias: Begg'stest, *P* = 0.051; Egger's test, *P* = 0.13. **(C)** Forest plot of hazard ratio (HR) of OS in comparison of LD-RT subgroup (>50.4 Gy) to HD-RT subgroup (>50.4 Gy) for SCC. CI, confidence interval; HD, high dose; LD, low dose; RT, radiotherapy.

For patients diagnosed with SCC, patients in the HD-RT group had a significantly better OS (pooled HR = 0.75, 95% CI = 0.69–0.81, *P* < 0.0001; Figure 3A). The evidence was consolidated when received ≥circa 60 Gy (pooled HR = 0.74, 95% CI = 0.68–0.80, *P* < 0.0001; Figure 3B). However, less strength of OS benefits was observed when comparing ≤ 50.4 with >50.4 Gy for SCC (pooled HR = 0.89, 95% CI = 0.67–1.19, *P* = 0.44; Figure 3C).

### Effect of Radiation Dose on Recurrence

**Figure 4:**
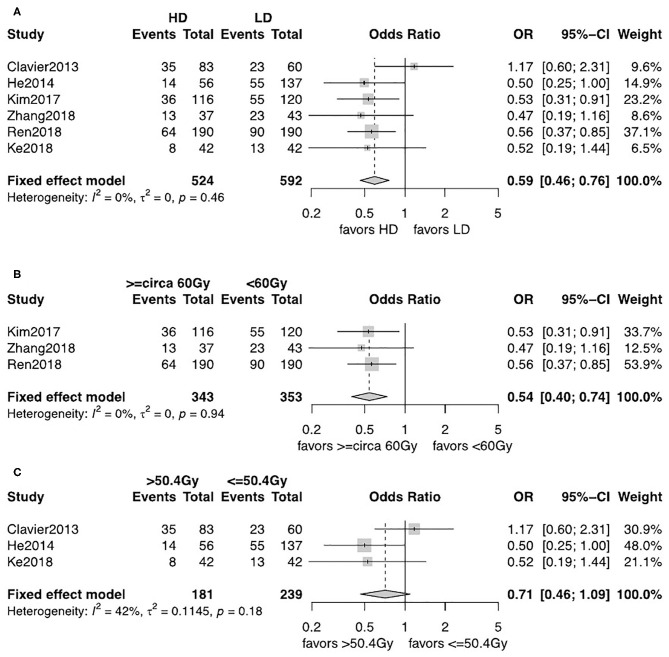
**(A)** Forest plot of odds ratio (OR) of LRF in comparison of LD-RT group to HD-RT group; Publication bias: Begg's test, *P* = 0.85; Egger's test, *P* = 0.95. **(B)** Forest plot of odds ratio (OR) of LRF in comparison of LD-RT subgroup (<circa 60 Gy) to HD-RT subgroup (≥circa 60 Gy). **(C)** Forest plot of odds ratio (OR) of LRF in comparison of LD-RT subgroup (≤ 50.4 Gy) to HD-RT subgroup (>50.4 Gy). Cl, confidence interval; HD, high dose; LD, low dose; RT, radiotherapy.

**Figure 5:**
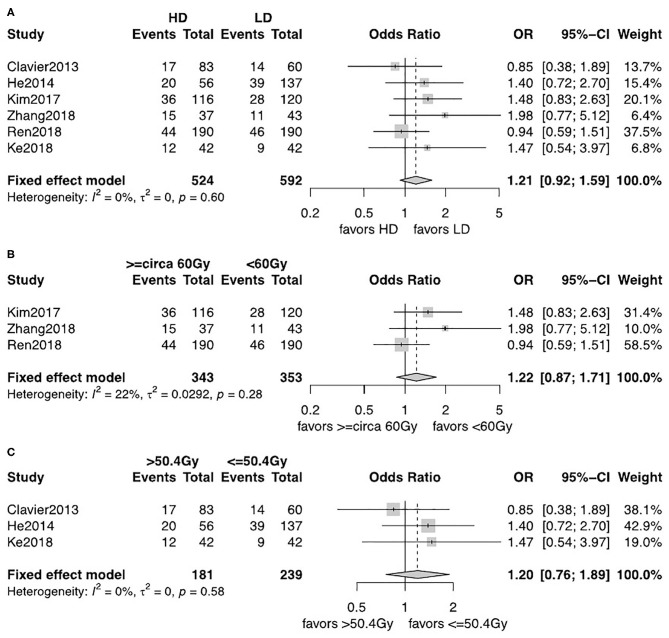
**(A)** Forest plot of odds ratio (OR) of DM in comparison of LD-RT group to HD-RT group; Publication bias: Begg's test, *P* = 0.57; Egger's test, *P* = 0.33. **(B)** Forest plot of odds ratio (OR) of DM in comparison of LD-RT subgroup (<circa 60 Gy) to HD-RT subgroup (≥circa 60 Gy). **(C)** Forest plot of odds ratio (OR) of DM in comparison of LD-RT subgroup (≤ 50.4 Gy) to HD-RT subgroup (>50.4 Gy). Cl, confidence interval; HD, high dose; LD, low dose; RT, radiotherapy.

The observed frequency of LRF and DM data was reported in six studies including 1,116 patients (12, 13, 18–21). A fixed-effects model was used after assessment of heterogeneity. LRF was significantly lower in the HD-RT group compared with the LD-RT group (OR = 0.59, 95% CI = 0.46–0.76, *P* < 0.0001; Figure 4A). There was no significant difference in the comparison of DM rate between the two groups (OR = 1.21, 95% CI = 0.92–1.59, *P* = 0.17; Figure 5A). No publication biases for the estimate of recurrences were detected. Similar results were found in subgroup analysis per cutoff. The patients receiving ≥circa 60 Gy radiation had a significant better local–regional control than those receiving <circa 60 Gy radiation (OR = 0.54, 95% CI = 0.40–0.74, *P* < 0.0001; Figure 4B). In contrast, in the studies comparing the ≤ 50.4 Gy subgroup with the >50.4 Gy subgroup, the evidence is statistically insignificant (OR = 0.71, 95% CI = 0.46–1.09, *P* = 0.18; Figure 4C). In addition, the subgroup analysis showed that neither the <circa 60 Gy nor the ≤ 50.4 Gy subgroup was significantly associated with less DM (OR = 1.22, 95% CI = 0.87–1.71, *P* = 0.26; Figure 5B and OR = 1.20, 95% CI = 0.76–1.89, *P* = 0.43; Figure 5C).

### Effect of Radiation Dose on Toxicities

The most common radiation-related acute toxicities of grade 3 or higher for esophageal carcinoma with CCRT were pneumonitis and esophagitis. The incidence and severity of treatment-related grade 3–5 toxicities are presented in Table 2. The pooled analysis results revealed that high radiation dose did not increase the risk of grade 3–5 pneumonitis (OR = 1.05, 95% CI = 0.54–2.05, *P* = 0.89), esophagitis (OR = 1.40, 95% CI = 0.93–2.11, *P* = 0.11), or treatment-related death (OR = 1.60, 95% CI = 0.70–3.66, *P* = 0.27) compared with low radiation dose. On the whole, escalated radiation dose did not increase the toxicities. Most of the patients could tolerate the toxic reactions.

**Table 2:** Adverse events of grades 3–5.

**Studies**	**Sample** **Size (LD/HD)**	**Radiation** **Technology**	**Radiotherapy Dose**		**Pneumonitis**	**Esophagitis**	**Treatment-Related** **Death**	**Evaluation Criterion** **for Toxicities**
			**LD-RT**	**HD-RT**				
(11)	24/20	IMRT	60 Gy/2 Gy	63.9 Gy/2.27 Gy	0 vs. 0	0 vs. 0	0 vs. 0	CTCAE 3.0
(12)	60/83	3D-CRT	38–50.4 Gy/1.8–2 Gy	50.7–72 Gy/1.8–2.5 Gy	0 vs. 0	6.8 vs. 8.4%	0 vs. 0	CTCAE 3.0
(13)	137/56	3D-CRT/IMRT	41.4–50.4 Gy/1.6–2.4 Gy	52.2–66 Gy/1.6–2.4 Gy	6.6% vs. 0	20.4 vs. 17.9%	5.1 vs. 3.6%	CTCAE 3.0
(17)	63/74	3D-CRT/IMRT	50–50.4 Gy/1.8–2 Gy	≥59.4 Gy/1.8–2 Gy	4.0 vs. 6.0%	2.2 vs. 10.5%	3.2 vs. 6.8%	CTCAE 3.0
(18)	120/116	3D-CRT/IMRT/ TOMO	<60 Gy/1.8–2 Gy	≥60 Gy/1.8–2 Gy	2.5% vs. 0	6.7 vs. 6.0%	1.7 vs. 0.9%	CTCAE 4.0
(19)	42/42	IMRT/VMAT	44–50.4 Gy/–	52.2–70 Gy/–	0 vs. 0	0 vs. 0	0 vs. 0	CTCAE 4.0
(20)	190/190	3D-CRT/IMRT	50.4–54 Gy/1.8 Gy	60 Gy/2 Gy	1.6 vs. 1.6%	2.6 vs. 7.4%	0.5 vs. 3.2%	CTCAE 4.0
(21)	43/37	3D-CRT/IMRT	50.4–56 Gy/1.8–2 Gy	59.4–64.8 Gy/1.8–2 Gy	9.3 vs. 27.0%	9.3 vs. 21.6%	0 vs. 0	CTCAE 4.0

*LD, low dose; HD, high dose; RT, radiotherapy; 3DRT, three-dimensional conformal radiotherapy; IMRT, intensity-modulated radiotherapy; VMAT, volume-modulated arc therapy; TOMO, helical tomotherapy; LD-RT vs. HD-RT group; CTCAE, Common Terminology Criteria for Adverse Events*.

## Discussion

For patients with esophageal cancer receiving CCRT, the recommended radiation dose remains controversial. Although several meta-analyses were published regarding the optimal radiotherapy dose of CCRT for esophageal carcinoma (26–28), the robustness of their findings was inadequate due to the limited sample size. Moreover, some outdated radiation techniques, including the improper multiple field technique, cobalt-60 equipment, and 2D-CRT, were included in these studies, introducing more heterogeneity. Thus, we performed an up-to-date meta-analysis to evaluate the clinical benefit of different radiation doses using modern radiation techniques in CCRT for esophageal carcinoma. The methodological quality was assessed to make sure that all studies included were scientifically conducted. This guaranteed the result's reliability of our study.

Overall, our meta-analysis based on 10,896 patients suggests that escalating radiation dose under modern radiation techniques could induce a favorable benefit–risk profile. The subgroup analyses in our study showed that ≥circa 60 Gy can significantly improve the OS as well as the local–regional control as compared with <circa 60 Gy, while >50.4 vs. ≤ 50.4 Gy showed no significant benefits for OS or local–regional control. The different results between the two subgroup analyses can be partially explained by the theory that 45 to 50-Gy radiation dose can be used to control microscopic tumors of SCC or AC, and at least 60 Gy is required aiming to control gross tumor (29–31). Our analysis is also in accordance with some nearly published studies (26, 32). The results suggest that when the dose of radiotherapy is raised within a certain range below the 60 Gy dose threshold, it would not improve the OS and local–regional control of esophageal carcinoma patients.

The previous study supports that the clinical features and biological behaviors are different between SCC and AC (33); the optimized radiation dose may also be influenced by the histology type. In our study, SCC accounted for 64.7% of all cases and 9 of the 12 studies included SCC patients only. In order to verify the effect of histology type, subgroup analyses based on histology type were also introduced. In the subgroup analyses, we found that patients diagnosed with SCC had a significantly better OS in the HD-RT group, especially for ≥circa 60 Gy. Pooled analysis with AC was not performed due to the lack of eligible study which only enrolled patients diagnosed with AC. Radiation-related toxicities may influence the survival benefits by high-dose radiation. Despite that the INT 0123 study failed to demonstrate the increased radiation dose could improve OS and local–regional control, investigators debate the burden of outdated radiation techniques which may under estimate the benefit. With the clinical application of more precise radiotherapy techniques such as 3D-CRT, IMRT, and VMAT, Welsh et al. (9) and Fakhrian et al. (34) reported that modern techniques could deliver higher doses to locally advanced esophageal carcinoma without increase in toxicities to the organ at risks (OARs), such as the heart, lung, liver, and spinal cord. Early results from a single-arm prospective phase II trial showed that using IMRT simultaneous integrated boost to escalate dose to 63 Gy for GTV of esophageal carcinoma was feasible with tolerable toxicities (32). Moreover, several retrospective studies support dose escalation in definitive CCRT with modern radiation techniques for esophageal carcinoma (6, 13, 14, 35, 36). Our meta-analysis showed that although esophagus-related toxicity and pulmonary injury were the main severe late toxicities, high radiation dose did not increase the toxicities, which finally convert to the survival benefit.

Two nearly closed RCTs reported the initial results by conference abstracts, which could not be included in this study (37, 38). Xu et al. (37) showed no difference toward 1 and 2 y-OS or treatment toxicity between the HD-RT (60 Gy) and LD-RT (50 Gy) groups. This result may be caused by the low radiotherapy completion rate in the HD-RT group (87.5 vs. 95.4%, *P* = 0.002). Moreover, the result renewed at the 2018 Chinese Society for Radiation Oncology (CSTRO) annual meeting showed a marked but nonstatistically significant improvement of 3 y-OS (63.1 vs. 55.7%, *P* = 0.199), which may be due to the limited sample size. The ARTDECO study (38) reported that the HD-RT (61.6 Gy) group did not result in a better OS or local–regional control than LD-RT (50.4 Gy) group. However, the radiation dose escalation was only delivered to the primary tumor with a numerical improvement of the local–regional control. Final conclusions cannot be drawn before the detailed data can be published.

Inevitably, there are some limitations in our analysis. Firstly, except one RCT and four population-based propensity-score-matched analyses, the other studies included were all retrospective ones, especially several studies only had a small sampling of patients. Moreover, our study is based on published data instead of individual patient data. This may reduce the comprehensiveness of the conclusion. Secondly, the heterogeneity of tumor stage, dose distribution, chemotherapy regimens, and radiotherapy volumes in different studies would confound the final results. Some of the data were absent in the included studies, it was difficult for us to evaluate the influence of these factors. Thirdly, our study was based on initially diagnosed esophageal carcinoma treated with definitive CCRT. The conclusion should be interpreted cautiously in radiotherapy alone, in sequential chemotherapy and radiotherapy, or in relapsed diseases.

## Conclusion

For patients with esophageal carcinoma receiving CCRT with modern radiation techniques, high-dose radiotherapy induces a favorable benefit–risk profile by improving the OS and local–regional control without increase in severe toxicities compared low-dose radiotherapy, especially in the ≥circa 60 Gy group vs. <circa 60 Gy group. However, the result should be interpreted cautiously before more prospective large-scale phase III randomized clinical trials can draw a definite conclusion.

## Data Availability Statement

All datasets presented in this study are included in the article/Supplementary Material.

## Author Contributions

XS and LW contributed substantially to the conception and design of the study, acquisition of data, quality control, data analysis and interpretation, statistical analysis, manuscript preparation, editing, and review. YW contributed to the design and statistical analysis of the study. JK, WJ, and YM contributed substantially to the quality control of data and review. ZH contributed substantially to the conception of the study, manuscript preparation, editing, and review. All authors contributed to the article and approved the submitted version.

## Conflict of Interest

The authors declare that the research was conducted in the absence of any commercial or financial relationships that could be construed as a potential conflict of interest.

## Supplementary Material

Supplementary Table 1 The quality of the included studies assessed by 9-star NOS.

Supplementary Table 2 The quality of the included studies assessed by modified Jadad Score.
