# Supplementary material for: High vs. Low Radiation Dose of Concurrent Chemoradiotherapy for Esophageal Carcinoma With Modern Radiotherapy Techniques: A Meta-Analysis
Source: Front Oncol. 2020 Aug 4;10:1222. doi: 10.3389/fonc.2020.01222 (PMC7418493; doi:10.3389/fonc.2020.01222)
Supplement: Supplementary Table 1 — The quality of the included studies assessed by 9-star NOS. [file Data_Sheet_1.pdf]

Supplementary Table 1. The quality of the included studies assessed by 9-star NOS

Study	Selection				Comparability	Exposure			Scores
	Adequate definition of cases	Representativeness of cases	Selection of controls	Definition of controls	Control for important factor	Ascertainment of exposure	Same method to ascertain for cases and controls	Non-response rate	
Clavier et al,2013(15)	☆	☆	☆	☆	☆	☆	☆	-	7
He et al, 2014(16)	☆	☆	☆	☆	☆	☆	☆	-	7
Brower et al, 2016(17)	☆	☆	☆	☆	☆	☆	☆	-	7
Chen et al, 2016(18)	☆	☆	☆	☆	☆☆	☆	☆	-	8
Chang et al, 2017(19)	☆	☆	☆	☆	☆	☆	☆	-	7
Kim et al, 2017(20)	☆	☆	☆	☆	☆	☆	☆	-	7
Deng et al, 2017(21)	☆	☆	☆	☆	☆	☆	☆	-	7
Zhang et al, 2018(22)	☆	☆	☆	☆	☆	☆	☆	-	7
Ren et al,2018(23)	☆	☆	☆	☆	☆☆	☆	☆	-	8
Ke et al, 2018(24)	☆	☆	☆	☆	☆	☆	☆	-	7
Li C et al, 2019(25)	☆	-	☆	☆	☆	☆	☆	-	6

NOS: Newcastle-Ottawa Scale.

Supplementary Table 2. The quality of the included studies assessed by modified Jadad Score

Studies (Year)	Randomization	Concealment of allocation	Double blinding	Withdrawals and dropouts	Score
Zhu et al, 2012(14)	1	2	2	0	5
